# Reframing preventive health communication: from individual compliance to collective action

**DOI:** 10.3389/fpubh.2026.1814344

**Published:** 2026-05-28

**Authors:** Amrita Misra, Putul Thakur

**Affiliations:** Project Concern International, Delhi, India

**Keywords:** health, JEEViKA, NRLM, nutrition, public health, SbcC, socio-ecological model, women’s collectives

## Abstract

Behaviour change in public health is a complex process that requires a comprehensive, contextually grounded social and behaviour change (SBC) approach, supported by effective health promotion platforms that move beyond individual-centric models towards collective social action. This paper presents learnings from the large-scale JEEViKA Technical Support Programme implemented in Bihar, which leverages women’s collectives as a health promotion platform and delivers context-specific messages based on an in-depth understanding of community barriers. The study adopts the case study method and reviews programme documents, research papers, monitoring and evaluation data and observational and experiential learnings, to analyse and document the learnings. Findings indicate that a Socio-Ecological Model-based SBC strategy implemented through women’s collectives significantly improved health and nutrition outcomes. Three key insights emerge: first, contextually grounded messages aligned with community practices are more readily adopted, whereas linear, didactic IEC approaches often remain limited to awareness generation; second, women’s collectives function as powerful platforms for social transformation; and third, linking communities with entitlements reinforces behaviour change. The study concludes that this integrated, multi-touchpoint approach offers a scalable model for addressing broader public health challenges, particularly non-communicable diseases.

## Background

India’s health landscape is burdened by several complex diseases. These include tuberculosis, malaria, diarrhoea, maternal and neonatal disorders, lymphatic filaria, hypertension, diabetes, obesity, mental health disorders and cancer among others (1). The Government of India (GoI) has launched several health initiatives, including Ayushman Bharat, the National Health Mission, Anaemia Mukt Bharat, Poshan Abhiyanand many other programmes to address these public health challenges. The success of these programmes depends not only on addressing systemic issues, but also on the adoption of contextually grounded health promotion and behaviour change strategies (2–4). Lessons from past epidemics and pandemics demonstrate that Social and Behaviour Change (SBC) approaches play a crucial role in social mobilisation, community engagement and risk communication (5). SBC is a strategic approach that promotes positive changes in knowledge, attitudes, norms, and behaviours by using audience segmentation, pre-tested messages, and a mix of mass media and interpersonal communication channels (6, 7). SBCs are most effective when they incorporate audience segmentation and tailored messaging, transparent risk communication, strong community engagement, prioritisation of key behaviours, and sustained political will and commitment (8–11). The effect of health behaviour intervention also depends upon the medium or platform used for its promotion, such as community institutions, digital platforms, social media or interpersonal communication (IPC) (12–14). Evidence also revealed that a successful health promotion extends beyond linear clinical health education delivered by doctors and frontline workers; it encompasses a range of individual, social, cultural, and economic determinants that influence behaviour change and its sustainability (15). The present community case study presents learnings from the decade-long “JEEViKA Technical Support Programme (JTSP) being implemented in Bihar”, which utilised community platforms of women’s collectives to develop people-centric social and behaviour change communication plans based on a thorough understanding of barriers to improve health and nutrition outcomes. Anchored in the Socio-Ecological Model, this multi-touchpoint approach promoted behaviour change not only at the individual level but also across families and the wider community, ultimately contributing to shifts in social norms and improved health outcomes. Thus, the objectives of this paper is to (i) describe the process of adapting and operationalising an SEM-based framework within women’s collectives, (ii) analyse its role in improving health and nutrition outcomes among mothers and children under 2 years of age, and (iii) generate actionable insights on leveraging community institutions as platforms to address several public health issues including communicable and non-communicable diseases to achieve scalable and sustainable behaviour change. By doing so, the paper contributes to ongoing discourse on people-centred public health approaches and offers a pathway for strengthening community-driven interventions towards achieving Universal Health Coverage.

## Rational for innovation

Many public health programmes in India continue to rely on a linear health promotion approach, wherein frontline workers such as ANMs (Auxiliary Nurse Midwives) and ASHAs (Accredited Social Health Activists) are tasked with delivering health messages to last-mile populations (16, 17). However, this didactic approach of health promotion faces several limitations, including issues related to incentive structures of health workers, caste dynamics, limited community support, gaps in health workers’ knowledge on specific topics, the geographical dispersion of households, and the influence of rigid socio-cultural norms (18–21). Moreover, linear, one-way communication models have been critiqued for their limited ability to address complex behavioural determinants, underscoring the need for more participatory and community-driven approaches (22, 23). Evidence suggests that programmes built on a socio-ecological model-based health promotion strategy can significantly reduce the burden of disease by addressing key social, behavioural, and structural determinants of health (24, 25). Despite this growing body of evidence, there remains a critical gap in understanding how community-based platforms, particularly women’s collectives, can be systematically leveraged to design and implement scalable, context-responsive SBC strategies. While these collectives have demonstrated potential in improving livelihoods and fostering social empowerment, their role as structured platforms for influencing health and nutrition behaviours, especially through integrated, theory-driven approaches such as the socio-ecological model (SEM), remains underexplored. Therefore, clearly articulating the process of integrating the Socio-Ecological Model (SEM) framework into public health programmes that leverage community platforms (like women’s collectives) across India is critical for achieving equitable universal health coverage. The community case study outlines the steps of integrating SEM framework into the community platforms for improving health outcomes.

## Context for innovation

The JEEViKA Technical Support Programme (JTSP) was implemented in the eastern state of India, Bihar. It is the third most populous state in India, and faces persistent challenges related to the poor nutritional status of young children, pregnant women and lactating mothers (26, 27). The JEEViKA initiative was launched in 2007 in Bihar with a clear mandate to enhance women’s economic empowerment by fostering livelihoods. However, it soon became evident that achieving sustainable livelihood outcomes required simultaneous attention to underlying health and nutrition challenges. The JEEViKA Technical Support Programme (JTSP) was implemented between 2015 and 2025, provides evidence that community women-driven health promotion strategies grounded in local contextual realities can improve health and nutrition outcomes. The approach leverages multiple, context-specific channels of health promotion informed by community needs and lived experiences, as well as the inherent capacity of women’s collectives to influence entrenched social norms and enable behaviour change through supportive environments, rather than relying solely on didactic, health worker–led messaging. As the intervention strategy was rolled out across Bihar, more and more women’s groups began engaging with the issue of nutrition. Between 2021 and 2023, a full-scale intervention across Bihar reaching 60,000 village organisations, known as the Family Dietary Diversity Campaign, was undertaken by JEEViKA.

## Methods

This study adopts a community case study approach to document and analyse the design, implementation, and outcomes of a large-scale Social Ecological Based-Behaviour Change Communication (SBCC) strategy implemented through women’s collectives in Bihar. The community case study design can be defined as a description of, and reflection upon, a programme or practice geared towards improving the health and functioning of a targeted population (28, 29). A case study design was considered appropriate given the complexity of the intervention, scale and geographic spread, its integration within real-world programmatic settings to address public health challenges while generating contextually grounded insights into behaviour change processes and outcomes. Several studies have been conducted in the past; however, those studies measured the outcome level change only (30–32). The study primarily aims to contribute to practice-based evidence, with a focus on understanding the process of identifying barriers and reframing contextually grounded SBC strategies leveraging potential community platforms. This case study is based on a triangulation of multiple data sources, including:

- Programme documents, design frameworks, and implementation guidelines
- Monitoring and evaluation data from the intervention period (2015–2025)
- Secondary data sources such as independent evaluations (published research papers by World Bank, Project Concern International, etc.)
- Observational insights and experiential learnings from programme implementation

A framework-based narrative approach was used to interpret and present findings. It includes insights identifying key barriers, developing new programmatic strategies to address those barriers, and pathways of change across multiple levels—individual, household, community, and system.

## Learnings from the “The JEEViKA Technical Support Programme”

### Identifying behavioural barriers to improve health and nutrition practices

Findings from consecutive rounds of the National Family Health Survey (Rounds 1 to 3) underscored the persistent and multifaceted health and nutrition vulnerabilities faced by pregnant and lactating women and children below 2 years of age in Bihar. However, a significant improvement was observed in the nutritional status of young children in NFHS round-5 —particularly in the reduction of stunting—as well as in key health indicators among women (27). These gains can be attributed to multiple initiatives undertaken by the Government of Bihar (GoB), along with efforts implemented through the women’s collectives’ platform under the JTSP programme.

The programme aimed to improve health and nutrition practices among pregnant and lactating women and children under 2 years of age by adopting a scientific Social and Behaviour Change (SBC) approach. Prior to the application of any behaviour change theory–based framework, it is essential to develop a comprehensive understanding of the contextual barriers and triggers influencing these practices.

Initially, under the JTSP programme, the strategy relied on a linear, module-based messaging approach. The target audience was women SHG members, adult women belonging to poor rural families, who are partially literate and have joined state-supported SHGs under the JEEVIKA programme for livelihood enhancement. The messaging was delivered during weekly meetings through trained peers designated as Community Mobilisers (CMs). These CMs underwent multiple rounds of modular training on maternal, infant and young child nutrition (MIYCN), and were incentivised for their effort. Messages conveyed by using methods such as pictorial Flip Book, mixed digital media, interactive games and food demonstrations included information on diverse food groups and their consumption practices during pregnancy, lactation and complementary feeding. While the targeted intervention demonstrated positive results at the pilot level, its scale-up revealed several structural and normative barriers. These early experiences highlighted the limitations of relying on traditional, linear Information, Education, and Communication (IEC) approaches to drive sustained behaviour change at scale. Despite the intrinsic strength of the SHG platform, entrenched socio-economic and cultural constraints limited the teams’ ability to meaningfully introduce and position maternal and child nutrition as a salient issue within the group. Firstly, it was found that preventive and promotive health and nutrition occupy low priority in rural households, where livelihood insecurity, food availability, and domestic responsibilities assume precedence (33). Secondly, women do not perceive malnutrition as an urgent concern. Specifically, maternal and child nutrition is a family matter—pregnant women and children eat from the household pot—the act of cooking is conducted for the family as a whole, not for individual members of the family, including young children (34). Thirdly, innumerable social norms determine food practices of mothers and children, providing a false sense of security in their traditional knowledge and fourthly, each SHG has a diverse age composition of members, leaning towards ages above 35 years which created uneven engagement with the issue (35)—while women with infants and toddlers aligned with the issue; older women and grandmothers perceived it as irrelevant. Comments such as—“My children are grown up, this information is irrelevant to me”, from older women signalled a mismatch between programme framing and lived experience.

Lastly, poor awareness of nutrition practices, schemes, entitlements and other available systemic support and services created a sense of disempowerment among the group, making it difficult for them to visualise their role in improving maternal and child nutrition. The socio-ecological model–based SBCC approach adopted in the JTSP programme addresses these challenges by adopting a multi-touchpoint strategy for behaviour change that engages women, caregivers, husbands, mothers-in-law, communities, and policymakers simultaneously.

### The JEEViKA—a potential medium for driving health promotion

A potential and appropriate medium for SBC leads to transformative and sustained behaviour change. Within this context, women’s Collectives or Self-Help Groups have emerged as a crucial community platform and a central pillar of India’s poverty alleviation and social development architecture. Originating in the 1980s under the Integrated Rural Development Programme (IDRP), the SHG movement acquired scale and institutional coherence with the launch of the National Rural Livelihoods Mission (NRLM) in 2011, now implemented as the Deendayal Antyodaya Yojana–National Rural Livelihoods Mission (DAY-NRLM). Under this programme, over 10 million women’s collectives have been formed, bringing together more than 100 million rural women from economically marginalised households. Typically comprising 10–12 women from similar socio-economic backgrounds, these collectives meet regularly and engage in savings, credit, and collective decision-making (36).

Women’s collectives are recognised as critical spaces for social transformation and function through mechanisms described by the social identity model of collective action (SIMCA) (37). By transitioning women from isolated domestic roles into a cohesive social identity (38), the SHGs build collective efficacy—the shared belief that the group can successfully influence social and economic outcomes (39). This psychological shift is what enables a multi-pronged approach to succeed; when members identify with the collective ‘we,’ they are more likely to demand and manage shared resources like food security and health services. By leveraging the collective ‘we’ women’s collective platforms can move beyond simple awareness; they can provide multi-scalar “nudges,” fostering dialogue between family members, peers, community members and leaders, which is critical for changing community norms and behaviours. It is this capacity for sustained social discourse that can transform health from clinical instruction into a social development priority.

Recognising the potential of women’s collectives as a platform for social change, DAY-NRLM has leveraged this ecosystem to improve health and nutrition outcomes. While the core mandate of DAY-NRLM is poverty alleviation, poverty itself is multidimensional and closely interlinked with health and nutrition status. Secure and remunerative livelihoods enhance households’ ability to access diverse and nutritious foods, while good health and adequate nutrition are, in turn, essential for inclusive livelihood development and sustained productivity (40). Health, nutrition, and livelihoods therefore operate in a mutually reinforcing cycle, highlighting the need for integrated interventions that address these dimensions simultaneously. There is a growing body of evidence that demonstrate the effectiveness of SHGs in altering health and nutrition indicators (41–43) and influencing the social norms by collective decisions and strategic behaviour change approach (44). Findings of another study conducted in 2012, demonstrates significant improvements in key maternal and child health indicators achieved through interventions implemented via the SHG platform with an SBC approach, including increased uptake of antenatal care, institutional deliveries, and improved newborn care practices such as skin-to-skin care, dry cord care, early initiation of breastfeeding, and appropriate complementary feeding (45).

The pathways through which women’s collectives can effect change differ fundamentally from conventional public health strategies. The structured nature of the SHG platform provides programme implementers a gateway for simultaneous, repeated individual and collective interactions. Repeated nudges trigger community dialogues, encourage community activities and potentially involve community gatekeepers. Internal trust within SHG platforms provides safe spaces to women and a team approach to problem-solving promotes collective agency that fosters changes in social norms.

### Conceptual framework—reframing the behaviour change SBCC model

**Figure 1:**
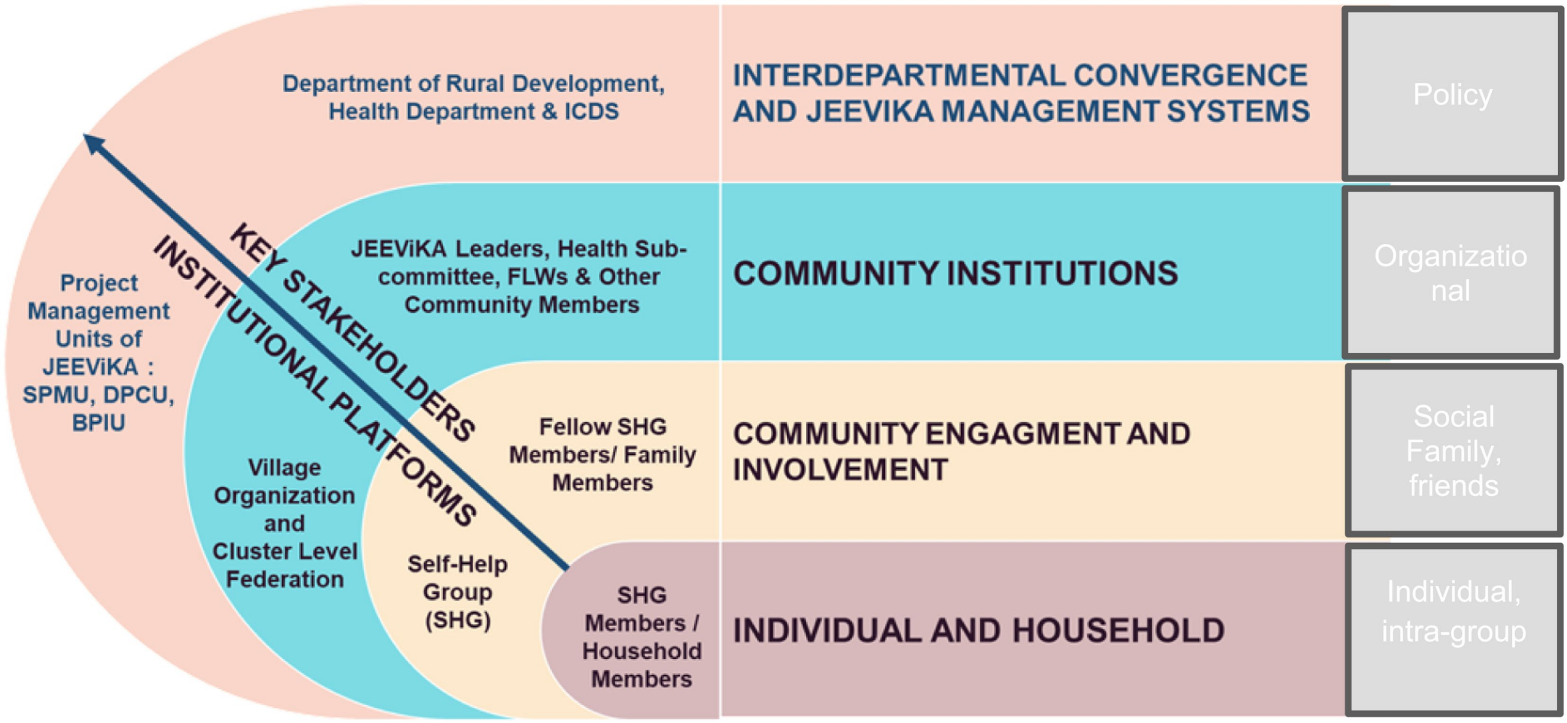
Socio-ecological model.

Recognising the interplay of personal, inter-group, social, cultural and organisational barriers to maternal and child nutrition, a holistic 360-degree Social and Behaviour Change Communication (SBCC) strategy based on the Socio-Ecological Model (SEM) was developed and pilot-tested leveraging JEEViKA’s platform. Figure 1 illustrates the architecture of the SEM of behaviour change communication.

A Human-Centred Design (HCD) process was utilised to understand the human factors behind low dietary diversity practices among women and children and to develop an effective intervention package that was both people-centred as well as evidence driven (45). The SBC strategy developed in consultation with programme implementers, SBC experts, SHG members and their leaders, drew on the findings of the HCD exercise to rethink the content of messaging for better community acceptance. The emerging JEEViKA SEM model dictated a shift from selective community messaging on maternal and child diet diversity to comprehensively mobilising each platform of the SEM architecture to focus on dietary diversity of the family as a whole. While including direct messages to improve diet diversity among mothers and children, the socio-ecological model included messages to address family dietary diversity as a whole, explaining the cross-cutting impact of poor nutrition across all aspects of daily living—financial draw on family income due to poor health due to malnutrition, low productivity, poor school performance, poor birth outcomes, lower participation in community activities were included, e.g., a weak malnourished family is unable to lead an economically productive life. Among target groups, this approach established good nourishment as intrinsic to individual, family and community well-being rather than being simply a mother and child specific issue. It removed age-barriers and normative barriers related to domestic cooking practices. Initially in the programme design, only SHG members were included as the target audience but, heeding the role of influencers—family and friends, community leaders and health workers in shaping nutrition narratives, the forum was opened for all. Multi-pronged approaches were used to disseminate the messages—messaging via interactive and participatory methods at multiple points, using a mix of household, community and digital engagements. The delivery channels were later expanded to include home visits, rallies, food and recipe demonstrations and reward and recognition programmes.

Simultaneously, project team promoted demand generation for schemes and entitlements through awareness generation and worked with systems to facilitate household access to schemes and entitlements that fortify household food security—PDS, THR, Mid-day meal. Technical assistance for creating a homestead nutrition garden, improved animal husbandry was facilitated through inter-departmental cooperation between the Health and Nutrition vertical with the Farm vertical of BRLPS.

This approach of holistic environment building around nutrition by demonstrating the intersectionality of nutrition with several aspects of life—economic, social and cultural and the visualisation of a pathway for action proved far more inclusive, drawing in women across age groups and household roles (32). The emphasis on ‘family diet diversity’ alongside ‘maternal and child diet diversity’ eliminated the need to change the socio-cultural practice of cooking for the family versus cooking separately for an individual, i.e., mother or child (47) which is a challenging activity for households with limited time and resources where it is the sole responsibility of women to cook for the entire family. The socio-economic model remained aligned with the programme’s objectives, as children’s dietary practices are shaped by overall household consumption patterns, making family-wide nutrition education a strategic entry point and the impact of nutrition on family life the pivot for action.

## Results

The JTSP programme is a large-scale initiative that has evolved over time by leveraging the strengths of the women’s collective community platform. Multiple studies conducted by various organisations have demonstrated the effectiveness of this platform in improving health and nutrition outcomes in Bihar. Table 1 summarises evidence from these studies, highlighting the efficacy of an SEM-based SBC approach delivered through the platform.

**Table 1:** Evidence demonstrating the efficacy of the SHG platform adopting SEM-based SBC approach.

Intervention through SHG platform	Name of the development partner	Year	Geography	Evaluation done	Findings
Gram Varta	BTAST/ DIFD	2012–16	35 blocks of 5 districts	Yes	Participants showed improved knowledge of healthy behaviours, and WASH, indicating potential gains in maternal and child health. The programme also enhanced women’s agency and decision-making in family health matters while challenging restrictive gender norms (56).
Multi-sectoral Convergence Program	IFPRI	2015–18	Three blocks of Saharsa district, Bihar	Yes	A cluster-randomised trial in Bihar showed that an SHG-based health and nutrition intervention improved service utilisation, knowledge, and key behaviours. Over 2.5 years, it increased children’s dietary diversity by 7% and women achieving minimum dietary diversity by 30%, though no impact was observed on BMI or child undernutrition (57).
Swabhimaan	UNICEF	2015		Yes	Community-led interventions targeted 18 health and nutrition practices. Primary outcomes focused on reducing undernutrition (BMI < 18.5) among adolescent girls and mothers and improving maternal nutritional status (MUAC) among pregnant women (48).
Parivartan	Project Concern International	2011–15	9,000 SHGs across 8 administrative blocks	Yes	A study in Bihar (2012–2017) examined SHG-based Health Layering (HL) models (Parivartan and JEEViKA+HL) and found significant improvements in RMNCHN and sanitation behaviours. About 64% of indicators were higher among Parivartan members, and during scale-up, 50% of indicators showed better outcomes among health-layered SHG members compared to those without health layering (58).
BTDP	World Bank	2015–2024	345 blocks	Yes	Significant improvements in dietary practices were observed, with dietary diversity among children (6–23 months) increasing from 8 to 53% and among women from 9 to 54% across evaluation rounds. These findings demonstrate the effectiveness of JEEViKA’s multi-sectoral convergence approach in improving nutritional outcomes (59).
JTSP	Project Concern International	2015–2024			An external evaluation by the Population Council (2017–2018) demonstrated the effectiveness of the Navratna tool based BCC approach delivered through SHGs, home visits, community events, and convergence with frontline workers. The intervention led to significant improvements in key practices, including timely initiation of breastfeeding (DID: 9.4 percentage points [pp], *p* < 0.05), exclusive breastfeeding (DID: 13.0 pp., *p* < 0.001), child dietary diversity (DID: 12.9 pp., *p* < 0.001), minimum meal frequency (DID: 24.9 pp., *p* < 0.001), and minimum acceptable diet (DID: 16.1 pp., *p* < 0.001) (60).

The demonstrated impact prompted Bihar to scale the SBCC strategy statewide across all 534 blocks, and its subsequent incorporation into the National Food, Nutrition, Health and WASH (FNHW) strategy under DAY-NRLM. The adoption of the socio-ecological SBCC model across all states—covering over 7 lakh villages and more than 10 million SHGs—reflects both its institutional feasibility and policy relevance. Beyond nutrition, the experience suggests that women’s collectives with the defined SBCC strategy can serve as effective platforms for addressing other multidimensional development challenges and public health issues, provided interventions are designed to engage social norms, collective agency, and systemic enablers simultaneously.

## Discussion

The study presents learnings from a large-scale programme on framing SBC strategy to improve health and nutrition outcomes. The study presents three salient findings and recommends framing SBC strategies to address larger public health challenges. First, the contextually grounded messages based on community practices are adopted easily by the community in the Indian context, preventive and promotive health messages are reached to communities through defined Information Education and Communication (IEC) strategies—each public health programme has an IEC strategy. Community-facing messages prescribed in IEC strategies are usually designed adopting a medical disease avoidance lens, i.e., messages are medical, linear and individual-centric, e.g., eating calcium-rich foods to keep bones strong. Such messages are disbursed by field-level workers (ASHA, ANM) to a specific target audience. While this approach to messaging on health challenges has effectively capacitated health workers, improved disease awareness or nudged individuals for healthcare service uptake, it has rarely effectively mobilised communities for mass adoption of good health practices or created mass movement for health promotive action, e.g., banning high-sugar eatables near schools to reduce obesity or improved community demand for health services. A linear approach ignores the holistic impact of a disease on an individual and the community’s daily life and functions. A medical approach to health does not trigger discussions or debates; it limits a health problem as a solely ‘individual’ responsibility for health services to solve. Evidence from a programme aimed at reducing neonatal mortality in Shivgarh district of Uttar Pradesh, India highlights a persistent gap between the discovery of scientifically proven practices for improving newborn health and their adoption at the household level. The widening gaps observed between the scientific messages and community practices. This disconnect suggests that messaging alone—focused on preventive maternal and newborn care behaviours such as skin-to-skin contact and early initiation of breastfeeding, even when delivered through effective health worker–community interactions—is insufficient to drive sustained behaviour change. Therefore, there is a need to unpack the “black box” of community behaviours and to create pathways of least resistance that enable families to transition from existing practices to evidence-based behaviours (48). IEC succeeds when it is planned with a comprehensive strategy, i.e., clearly articulated objectives, keeping the community at the centre of what is being designed, conducting appropriate research, undertaking audience segmentation, integrating local perspectives, carefully crafting and testing messages, knowing and using appropriate channel choices, co-creating interventions with communities and planning for monitoring and feedback (49, 50). National flagship programmes often face limitations when relying on linear IEC and BCC strategies, particularly due to implementation challenges, constrained funding, and insufficient gains in community knowledge, reach and human resource (ASHA, AWW) capacity (51).

Secondly, women’s collectives offer distinctive institutional spaces for social transformation and sustained behaviour change. This article is an attempt to emphasise that to leverage the SHG platform effectively for health outcomes, the public health system should adopt a ‘person-centric’ rights-based approach to behaviour change, diverging from the traditional clinical IEC approach. By adopting a Socio-ecological approach, the JTSP succeeded in positioning nutrition as a critical factor for individual and community well-being, thereby promoting community ownership and action for better nutrition outcomes. In addition, an SBCC strategy operated through multiple and reinforcing touchpoints—individual, household, peer, community, and policy—thereby addressing behavioural, social, and structural determinants of nutrition simultaneously helped in message reinforcement. Several other studies also demonstrate the potency of the SEM framework through women’s collectives platforms for sustained social behaviour change and advocate its adoption to address public health issues (52, 53). And third, by mobilising systems to link households to food security schemes and social entitlements, the strategy extended behaviour change beyond awareness generation to the creation of enabling environments. The emphasis on family dietary diversity, rather than individualised feeding practices, aligned programmatic goals with lived realities of household cooking and consumption, facilitating wider acceptance across age groups and family roles. Moreover, POSHAN Abhiyan recognises SEM based SBCC as a critical driver of improved nutrition outcomes. The programme adopts a multi-touchpoint approach to behaviour change, engaging communities through a range of interventions including community-based events, intensive campaigns, awareness drives, and the use of digital media. These activities are systematically implemented each year during *Poshan Maah*, reinforcing key nutrition messages through repeated and complementary channels (54, 55). Thus, the paper strongly recommends adopting a comprehensive, contextually grounded, community practice–based approach that leverages community platforms such as women’s collectives to address a range of public health issues, including non-communicable diseases, obesity, hypertension, diabetes, and peri-menopausal concerns. The strategy goes beyond merely improving individuals’ knowledge; it also supports behaviour change by providing nudges at multiple touchpoints. The findings of this paper are largely drawn from experiences in Bihar, which may limit their generalizability to other regions with different socio-cultural and health system contexts. The analysis primarily focuses on health (particularly maternal health) and nutrition, and its applicability to other public health issues, such as non-communicable diseases, remains suggestive rather than empirically validated. Additionally, the paper does not fully account for broader system-level constraints, including resource limitations and variations in governance. Lastly, the effectiveness of the approach is highly dependent on the quality of facilitation, which may vary across settings.

## Conclusion

To improve health indicators and move towards ‘Viksit Bharat’ India needs to access all possible available resources. In the last decade or so, women’s collectives have demonstrated (in models) their capacity as an effective platform through which communities can be engaged to improve their own health indicators. However, to meaningfully mobilise collectives and bring about large-scale effective change, community engagement narratives of the public health sector should evolve beyond merely clinical discourses. Investing in more holistic SBCC strategies, such as a socio-ecological model, that is truly ‘person and community-centric’ would encourage communities to engage more meaningfully with health challenges. The community case story highlights several actionable insights for leveraging community institutions as platforms for addressing diverse public health challenges. First, embedding SBC strategies within existing community platforms enhances reach, acceptability, and sustainability by building on established trust and social capital. Second, operationalising multi-level frameworks such as the SEM enables interventions to address not only individual knowledge gaps but also structural normative and systemic barriers to behaviour change. Third, the integration of participatory and context-responsive approaches rooted in lived realities strengthens community ownership and facilitates collective efficacy, which is critical for influencing entrenched social norms. Finally, the platform-based approach demonstrates potential for scalability and adaptability across a range of public health issues, including communicable and non-communicable diseases, by leveraging existing institutional structures and community networks. This model offers a viable pathway for achieving sustained behaviour change at scale in resource-constrained settings.

## Data Availability

The original contributions presented in the study are included in the article/supplementary material, further inquiries can be directed to the corresponding author.
